# Drivers’ Visual Search Behavior Toward Vulnerable Road Users at Junctions as a Function of Cycling Experience

**DOI:** 10.1177/0018720818778960

**Published:** 2018-06-15

**Authors:** Chloe Jade Robbins, Peter Chapman

**Affiliations:** University of Nottingham, UK

**Keywords:** attentional processes, visual search, simulation, bicycle safety, eye tracking

## Abstract

**Objectives:**

The current study investigated the behavior and visual attention of two groups of drivers with differing pedal cycling experience (pedal cyclists and nonpedal cyclists) towards vulnerable road users at junctions in a driving simulator.

**Background:**

Pedal cyclists and motorcyclists are involved in a disproportionate number of crashes given the distance they travel, with a high proportion of these crashes occurring at junctions. Many studies have found that car drivers who also hold a motorcycle license have increased awareness towards motorcycles.

**Methods:**

The task involved approaching a T-junction and turning right when it was deemed to be safe. In Study 1, the junction was controlled by a give way sign, and in Study 2, the junction was controlled by a stop sign. Each T-junction contained a target vehicle (car, motorcycle, or pedal cycle), approaching from a near, medium, or far distance from the junction.

**Results:**

Participants did not look at pedal cycles approaching from a far distance for as long as they looked at approaching motorcycles and cars, despite all vehicles travelling at identical speeds. No differences were found between pedal cyclists and nonpedal cyclists on any visual attention measures, indicating that pedal cycling experience was not associated with differences in drivers’ attention toward pedal cycles.

**Conclusions:**

Findings have implications for road safety, demonstrating subtle differences in drivers’ everyday visual attention toward differing vehicle types.

**Applications:**

This research has the potential to inform the development of in-car technical assistive systems, improving the safety of vulnerable road users at junctions.

## Introduction

Motorcyclists and pedal cyclists are vulnerable road users, involved in a large number of road crashes. There has been a great increase in seriously injured motorcyclists and pedal cyclists in the United Kingdom over the last 7 years, with an estimated rise of 5% for motorcycles and 7% for pedal cycles at the end of 2016 compared with the 2010–2014 average (Department of Transport, 2016). This rise can be explained, in part, by the increasing motorcycle and pedal cycle traffic on the road; however, these road users are nonetheless involved in a disproportionate number of crashes given the distance they travel.

Motorcycle crashes have been studied in more depth than pedal cycle crashes. The most frequent type of motorcycle crash in the United Kingdom has been identified as ROW (right of way) crashes, whereby another road user pulls out of a side junction into the path of a motorcycle on a main carriageway (Clarke, Ward, Bartle, & Truman, 2007), also commonly termed the “look but fail to see” (LBFTS) error (Brown, 2002). It is typical in these crashes that drivers report being careful and attentive with their visual checks, but nonetheless they fail to see an oncoming road user. The majority of these crashes occur at “uncontrolled” (i.e., no stop light or sign with only give way markings and/or sign) T-junctions in urban environments (Hole, Tyrrell, & Langham, 1996).

In a more recent U.K. study, Pai and Saleh (2008) explored motorcycle injuries at T-junctions. It was found that injuries were the greatest when approaching motorcycles collided with a vehicle turning right, and injuries worsened when that junction was controlled by a stop or give way sign. Similarly, in regards to pedal cycles, Stone and Broughton (2003) extracted over 30,000 standardized reports from serious injury cycling crashes in the United Kingdom and found that one of the most frequent pedal cycle crashes at T-junctions occurs when the pedal cycle is travelling on a main road from the right and another vehicle is turning right onto the main road.

One of a few studies investigating drivers’ visual search towards pedal cycles investigated drivers’ selective attention at on-road intersections, using hidden video cameras to measure drivers’ head movements (Summala, Pasanen, Räsänen, & Sievänen, 1996; see also Räsänen & Summala, 1998). This was conducted on roads in Finland on which traffic drives on the right-hand side. It was found that the most prevalent pedal cycle crash occurs when the cyclist is coming from the right and a driver is pulling out of a side road and turning right. This was seen to be caused by inappropriate visual search strategies, with the driver scanning the right side of the intersection less frequently than the left side, presumably because drivers failed to give sufficient importance to traffic in the cycle lanes. This seems to be a different crash type to the one described earlier (Stone & Broughton, 2003); however, in this Finnish study, cyclists were travelling on a dedicated two-way cycle lane that the approaching vehicle had to cross before joining the main road. Such cycle lanes are rare on British roads, where most cyclists have to travel with the rest of the traffic. Crashes on British roads are thus more likely to be related to failures in attention towards cyclists when they are using the same road infrastructure as other vehicles.

The previous studies that examined drivers’ behavior and visual attention toward pedal cycles at junctions have investigated naturalistic events and accidents in order to capture drivers’ everyday on-road behavior. These studies used video validations and reconstructions in order to estimate the speed and distance of approaching vehicles in these instances, as these factors cannot be controlled. This makes it difficult to determine whether differences in visual search are because an approaching vehicle is a cyclist or are simply related to the speeds at which the vehicle is coming. By investigating drivers’ behavior and visual attention toward different road users in a simulated environment, it becomes possible to match the speeds and distances of different vehicle types, allowing for the investigation of drivers’ visual search toward differing vehicle types when they are approaching a junction at identical speeds.

In regards to experience, there have been studies showing that drivers who also ride a motorcycle have increased detection of motorcycles compared with drivers with no motorcycle experience. Magazzù, Comelli, and Marinoni (2006) conducted a case control study to investigate how motorcycle experience can affect crash risk. It was found that drivers who have a motorcycle license are less prone to be involved in car-motorcycle collisions compared with drivers with no motorcycle license. This suggests that the riding ability and the increased awareness of the dangers associated with motorcycles at junctions may help with the detection of oncoming motorcycles and the prediction of their maneuvers. Brooks and Guppy (1990) found that car drivers who have family members or close friends that ride motorcycles are also less likely to collide with motorcyclists and showed better observation toward motorcycles than drivers who did not.

Crundall, Crundall, Clarke, and Shahar (2012) investigated visual attention toward motorcycles by comparing experienced and novice drivers with “dual drivers” (car drivers with considerable experience of both car driving and motorcycle riding). Participants were presented with video clips, which displayed a car approaching and stopping at a junction. Participants were asked to imagine they were driving the car and had to press a button when they believed it was safe to pull out. Some clips contained an oncoming car, motorcycle, or no vehicles. It was found that experienced drivers’ fixation durations toward motorcycles were much shorter than those of “dual drivers” and novice drivers. Crundall et al. (2012) proposed that experienced drivers do not realize they are looking at a motorcycle and therefore terminate their gaze prematurely. This was attributed to overlearned visual search strategies and decreased expectations of approaching motorcycles. Dual drivers were seen as “gold standard” performers on all measures, suggesting that this group has an increased understanding that motorcycles require special attention. Whether or not the authors’ interpretation is correct, the findings clearly indicate that car drivers’ attentional allocation is strongly affected by motorcycle experience.

In a more recent study, Beanland and Hansen (2017) explored the influence of nondriving experiences on attentional allocation by comparing drivers with and without cycling experience. Twenty drivers and 22 cyclist-drivers were recruited to perform a change detection flicker task, with participants needing to determine whether two alternating images are identical or differ in one detail. Participants were instructed to imagine they were driving when viewing each road scene. The changed object was either a road sign, car, pedestrian, or bicycle. Cyclist-drivers were significantly faster at identifying changes, in particular to the road sign and bicycle. It was concluded that drivers with cycling experience have more efficient attentional processing of some aspects of road scenes.

In light of previous research, the two current studies investigate drivers’ visual search behavior toward pedal cycles and motorcycles in a high-fidelity driving simulator. We wanted to discover whether differences found in change-detection and video-based tasks could also be observed when drivers are freely controlling the vehicle. Critically we wanted drivers to make real decisions where they actually had to pull out at a junction to be sure that visual search strategies are representative of those used in real driving situations. In order to manipulate the likelihood of the driver actually pulling out in front of an oncoming vehicle, we added a naturalistic manipulation to the junction. This was added whether it was controlled by a “Give Way” sign (Study 1) or a “Stop Sign” (Study 2). It was our expectation that drivers would be more likely to wait for oncoming vehicles to pass if they knew that they had to actually stop at the junction; thus, Study 1 would provide details of visual search in situations where drivers generally pull out ahead of oncoming vehicles, while Study 2 would provide information about visual search in cases where the driver generally waits for an oncoming vehicle. These studies additionally investigate the effect pedal cycling experience has on drivers’ visual search at junctions, comparing pedal cyclist drivers to nonpedal cyclist drivers. Most previous research investigating drivers’ visual search toward pedal cycles have focused on either real on-road data or used static images of road scenes. The current studies thus have important implications for road safety, providing a better understanding of drivers’ different visual search toward oncoming vehicles approaching at identical speeds, focusing on road users with differing pedal cycling experience.

## Methods

### Participants

This research complied with the American Psychological Association Code of Ethics and was approved by the Institutional Review Board at The University of Nottingham. Informed consent was obtained from each participant.

Data were collected from 80 participants who received a £5 inconvenience allowance for their time. Forty participants took part in Study 1, which included a “Give Way” sign at the junction, and 40 participants took part in Study 2, which included a “Stop” sign at the junction.

Participants were recruited based on how often they used a pedal cycle. In Study 1, 20 pedal cyclists who held a driving license and cycled frequently (mean age = 24 years, *SD* = 6.8, range = 20–45; male = 11, female = 9) and 20 nonpedal cyclists who did not cycle frequently (mean age = 22, *SD* = 2.1, range = 20–28; male = 6, female = 14) were recruited. Pedal cyclists reported having held a driving license for between 1 and 264 months (*M* = 53.95), with a reported annual mileage between 60 and 15,000 miles (*M* = 3,668) and reported cycling for between 7 and 240 months (*M* = 78.12), with an average annual mileage of 624 miles. Nonpedal cyclists reported having held a driving license for between 8 and 120 months (*M* = 53.7), with a reported annual mileage between 50 and 10,000 miles (*M* = 2,699).

In Study 2, 20 pedal cyclists (mean age = 25 years, *SD* = 7.3, range = 20–45; male = 11, female = 9) and 20 nonpedal cyclists (mean age = 22, *SD* = 7.3, range = 19–45; male = 5, female = 15) were recruited. Pedal cyclists reported having held a driving license for between 3 and 252 months (*M* = 83.40), with a reported annual mileage between 50 and 10,000 miles (*M* = 3,460) and reported cycling for between 12 and 360 months (*M* = 162.13), with an average annual mileage of 680 miles. Nonpedal cyclists reported having held a driving license for between 10 and 120 months (*M* = 45.9), with a reported annual mileage between 50 and 10,000 miles (*M* = 3,327).

### Design

Although the two studies were conducted separately, to aid brevity in reporting, they are combined for analysis purposes. The two studies differed by the sign that was displayed at the entrance to the junction, in order to understand drivers’ behavior and visual search at “Give Way” and “Stop” controlled junctions. The experiments did not differ in any other way. A 2 × 2 × 3 × 3 mixed design formed the core of the combined analysis, with two between-subjects factors, which were Road Sign (Give Way vs. Stop) and Group (pedal cyclists and nonpedal cyclists), and two within-subjects factors relating to the oncoming target vehicles, which were Vehicle Type (pedal cycle, motorcycle, and car) and Distance (near, medium, and far).

**Figure 1.**
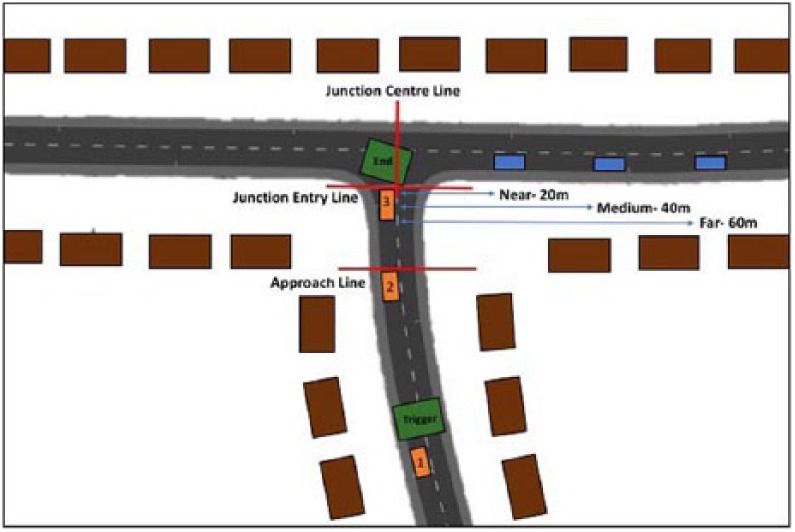
Parameters of the junction used in the experiment. The road and houses are to scale; however, the vehicles have been made larger to make them more visible. The participant’s vehicle in Position 1 shows the driver approaching the junction, just before entering the trigger box. This trigger box initiates the movement of the target vehicle. Position 2 indicates the point where the participant enters the approach zone. This is the point where the houses no longer occlude the junction, with the left- and right-hand side of the junction and the approaching traffic becoming clearly visible. Position 3 is the point where the driver has reached the junction entry line. Once the participant initiates a right turn maneuver and enters the “end” box, this terminates the trial. The junction center line is the line used to indicate a right or left fixation and determine whether this was toward or away from the approaching vehicle. The three vehicles positioned on the right-hand side of the junction indicate the typical near, medium, and far distance vehicles at the point where the driver has reached the junction entry line (Position 3). The green boxes represent trigger points programmed in the simulator, and the red lines were points defined by the experimenters in order to analyze results.

Each scenario started with the participant placed 135 m from the junction entry line; therefore, as participants were instructed to approach the junction at 20 mph, it took approximately 15 seconds to drive from the start position to the junction entry line. On the approach to the junction, participants drove over a trigger box, which was a point that triggered the target vehicle to start moving. The trigger box was 50 m from the junction entry line; therefore, it took approximately 5 seconds to drive from the trigger box to the junction entry line. At this point, no target vehicle was yet visible to the driver (see Figure 1). Once the participant had reached the junction entry line, the near, medium, and far distance target vehicles had always come into sight but differed in the amount of time it would still take them to reach the center of the junction, travelling at a speed of 15 mph (typically, near = 3 seconds, medium = 6 seconds, far = 9 seconds). This meant that the starting points of the target vehicles were 20 m, 40 m, and 60 m, respectively. It must be noted that these timings may differ slightly, as these depend on the exact approach and stopping behavior of the participant. Although it was technically possible to pass in front of the oncoming vehicle, attempting to do so when the vehicle was at a near distance did not normally make it possible to come to a complete halt at the junction before pulling out.

On the approach to the junction, the full junction and the target vehicles became visible approximately 20 m from the junction entry line, known as the start of the “approach zone”; therefore, it took approximately 2 seconds to reach the junction entry line. Before this point, the junction was occluded by houses on either side of the road (see Figure 1). In regards to the dynamics of the junction, more of the right-hand side of the junction was visible earlier on; however, as the right-hand side vehicles were approaching in the closer lane, they were initially less visible compared with the vehicles approaching from the left-hand side of the junction, in the further lane. As this is a naturalistic junction, whereby the right- and left-hand side of the junction are slightly different and therefore are imitating a real-life situation, any differences in drivers’ visual attention between left and right may be due to the specific parameters of the junction. For this reason, right and left traffic were not analyzed separately.

Each target vehicle (pedal cycle, motorcycle, and car) was placed at all three distances (near, medium, and far) and appeared from the left and right with equal frequency, all traveling at 15 mph. These scenarios only contained the target vehicle, with no other traffic. There were 18 experimental trials. As the target vehicle’s movement was trigged before the start of the “approach zone,” the participants always saw the vehicles moving.

A further 12 general traffic scenarios that had no target vehicles but included general traffic were included to ensure that participants did not always expect a target vehicle and were scanning for traffic on the left and right side of the junction. Each scenario terminated the moment the driver pulled out into the junction (irrespective of whether this was before or after the approaching vehicle crossed the junction). This scenario termination point was positioned after the participant had committed to the right-hand turn maneuver, still allowing for a crash to occur if he or she had pulled out unsafely.

All trials were fully counterbalanced, with six orders (A, B, C, D, E, and F), containing all 30 trials in a random order. Both studies included the same randomized orders, with seven participants in each study completing Orders A, B, C, and D and six participants completing Orders E and F.

### Stimuli and Apparatus

**Figure 2.**
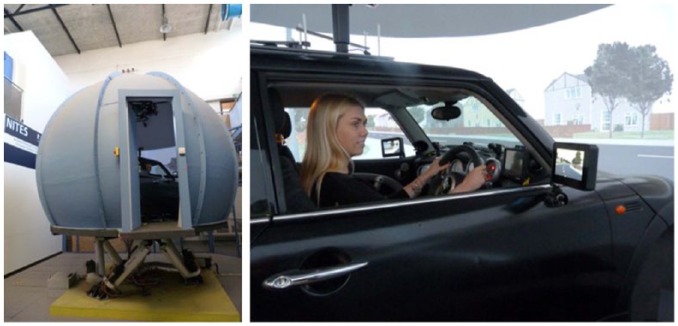
The NITES facility’s high-fidelity driving simulator. The simulator consists of a full BMW Mini, housed within a projection dome and mounted on a six-degree of freedom motion platform.

The experiment took place in the Nottingham Integrated Transport and Environment Simulation (NITES) facility’s high-fidelity driving simulator. This simulator comprises a full BMW Mini, housed within a projection dome and mounted on a six-degree motion platform with a 360-degree projection screen (see Figure 2). For the current studies, the motion base was turned off because the short trial lengths and abrupt terminations of each trial made the motion cues confusing. The scenarios were formed on the screens using six projectors. The simulator was equipped with two linked FaceLAB 5.0 eye-tracking systems (four cameras and two infrared sources), which allowed participants’ eye movements to be tracked continuously over a range of approximately 120 degrees in front of the driver.

**Figure 3.**
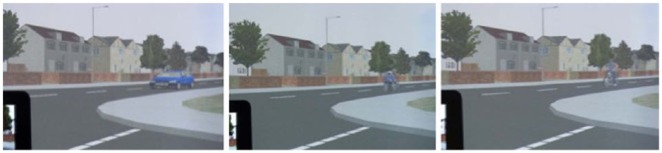
The three vehicle types used in the experiment (car, motorcycle, and pedal cycle). These are taken from the view of the driver, with the vehicles approaching from the right. These vehicles are approaching from a near distance.

XPI (XPI Simulation, London, UK) driving simulation software was used to create 30 scenarios. All scenarios took place at the same T-junction. As the experiment was conducted in the United Kingdom, all driving was conducted on the left-hand side of the roads. Figure 3 shows an example of all three vehicles used in the experiment (car, motorcycle, and pedal cycle) from the view of the driver, approaching from the right. These vehicles are placed at the near distance, from the point where the driver had reached the junction entry line. In regards to the pedal cycle, the simulated rider had a pedaling motion when moving.

### Procedure

Following a 5-minute practice drive, which was purposely more demanding than the experimental drive, participants completed a short “Driving & Cycling Experience” questionnaire with a main purpose of understanding how often the participant drove and cycled. The primary task was explained to every participant by the experimenter reading out the following systematic instructions:

In this experiment, you will encounter a T-junction 30 times. Your task is to drive up to the T-junction at a speed of 20 mph and perform a maneuver at the end of the junction when it is deemed to be safe. An audio message will instruct you to turn right prior to stopping at the junction. Once you start to pull out of the junction, the scenario will immediately end and the next one will begin shortly after. You must try and drive as naturally as possible throughout the experiment.

For Study 2, which included a Stop sign, there was a slight change in the instructions: “An audio message will instruct you to turn right when approaching the junction.”

For all junctions, the audio clip contained the instruction “at the junction, go right.” A right turn was used as this was a more difficult and balanced task, compared with the alternative left turn. In order to make a right turn, drivers had to scan for oncoming traffic from both the left- and right-hand side of the junction.

Participants’ eye movements were recorded throughout each scenario. Each scenario was around 20 seconds long, and the whole experimental procedure lasted around 40 minutes.

## Results

### Driving Experience and Age

Drivers’ reported experience and ages were subject to a 2 × 2 between-groups ANOVA with factors of Road Sign (Give Way vs. Stop) and Group (pedal cyclists vs. nonpedal cyclists). Drivers’ licensure (in months), annual mileage, and age range were subject to a log transformation due to positive skew. These analyses confirmed that there were no significant differences in drivers’ licensure between groups, *F*(1, 76) = .34, MSE = .14, *p* > .05, n^2^_p_ = .004) or experiments, *F*(1, 76) = .05, MSE = .14, *p* > .05, n^2^_p_ = .001; in drivers’ annual mileage between groups, *F*(1, 76) = .03, MSE = .57, *p* > .05, n^2^_p_ = .001, or experiments, *F*(1, 76) = 1.26, MSE = .57, *p* > .05, n^2^_p_ = .02; and in age between groups, *F*(1, 76) = 4.00, MSE = .01, *p* > .05, n^2^_p_ = .04, or experiments, *F*(1, 76) = .02, MSE = .01, *p* > .05, n^2^_p_ = .001.

### Data Analysis

Most behavioral and eye movement measures were subject to a 2 × 2 × 3 × 3 mixed design ANOVA with factors of Road Sign (Give Way vs. Stop), Group (pedal cyclists vs. nonpedal cyclists), Vehicle Type (pedal cycle, motorcycle, and car), and Distance (near, medium, and far). For the factor of Target Vehicle, two a priori orthogonal contrasts were specified. The first contrast compared data from pedal cycle trials with that of motorcycle and car trials together to assess any overall effect of cycling experience toward pedal cycles. The second contrast compared motorcycle trials with car trials to assess any overall effect between these two motor vehicles. For the factor of Distance, contrasts were specified that tested for linear trends in the data. Each target vehicle approached at each distance from the left and right of the junction at equal frequency; however, for the purpose of analysis, the vehicle direction was aggregated to increase the number of trials contributing to each cell.

### Behavioral Measures

Driver behavior was measured by looking at Approach Behavior. Approach Behavior was obtained by calculating how long it took drivers to travel through the “approach zone.” The “approach zone” started 20 m from the junction entry line and finished at the moment where the front of the drivers’ car had entered the junction by crossing the junction entry line. The “approach zone” thus started when the left and right side of the junction first became visible, the target vehicles were visible, and at the point where approaching traffic may start to alter the approach behavior of the driver.

#### Approach behavior

In regards to drivers’ approach behavior, a main effect of Vehicle Type was found, *F*(2, 152) = 3.54, MSE = 18.06, *p* < .05, n^2^_p_ = .05, with contrasts revealing a significant difference between pedal cycles compared with cars and motorcycles, *F*(1, 76) = 5.67, MSE = 31.23, *p* < . 05, n^2^_p_ = .07. Participants approached the junction faster when a pedal cycle was approaching compared with a car or motorcycle. There was also a main effect of Distance, *F*(2, 152) = 12.25, MSE = 19.74, *p* < .001, n^2^_p_ = .14, with contrasts revealing a linear trend, *F*(1, 76) = 17.19, MSE = 7.85, *p* < . 001, n^2^_p_ = .18. Participants approached the junction faster when vehicles were approaching from a closer distance—for cars (near = 10.32 seconds, medium = 10.39 seconds, far = 10.87 seconds), for motorcycles (near = 9.52 seconds, medium = 10.47 seconds, far = 10.64 seconds), and for pedal cycles (near = 7.32 seconds, medium = 9.93 seconds, far = 11.23 seconds).

There was also an interaction between Vehicle Type and Distance, *F*(4, 304) = 2.89, MSE = 36.37, *p* < .05, n^2^_p_ = .04, with contrasts revealing a linear difference between pedal cycles compared with cars and motorcycles, *F*(1, 76) = 13.18, MSE = 29.43, *p* < . 01, n^2^_p_ = .15. Participants approached the junction faster when vehicles are approaching from a nearer distance. The combination of these two main effects and the interaction highlights the finding that drivers approached the junction fastest when there was a pedal cyclist approaching from a near distance.

### Eye Movement Measures

With drivers making big rapid head movements and fixations at wide eccentricities, it was difficult to always be sure of the quality of the eye tracking. This was particularly problematic at wide eccentricities where fixations were often made toward the target vehicle, but because of calibration difficulties, we could not be sure that the target vehicle was actually fixated. If we had chosen to adopt a very strict criterion for determining whether a vehicle was fixated, there is a danger that we would falsely conclude that far vehicles were rarely fixated simply because calibration was poorer at wide eccentricities. Because of this, we adopted a very conservative approach, focusing on the broad direction of fixation (toward or away from the target vehicle, rather than requiring an unambiguous fixation on the vehicle) and choosing dependent variables that would not be systematically affected by differences in calibration quality between individuals. This approach has the additional advantage that the visual angle subtended by the target vehicle has no direct effect on whether a fixation is regarded as being on the vehicle. Choosing to aggregate between left and right approach directions also ensures that none of the reported differences can be influenced by differences in calibration quality for extreme left and right angles.

In regards to drivers’ visual attention at the junction, we calculated three main variables of interest: Proportion of Fixations, Proportion of Gaze, and Mean Fixation Duration. A custom-built MatLab script was used to automatically analyze drivers’ eye movements, with a fixation dispersion threshold of 0.1 of a radian for 100 ms, to regard a fixation to be in progress. The Proportion of Fixations was calculated by measuring the number of fixations toward and away from the target vehicle side of the junction. The tolerance for fixations toward the target vehicle was any fixation made to the side of the junction center line where the target vehicle was approaching (see Figure 1), after the participant had crossed the approach line, and the target vehicle was still approaching the junction. This did not include any fixations toward the target vehicle when the vehicle had crossed the junction center line. Fixations away from the target vehicle were any fixations made to the side of the junction center line where the target vehicle was not approaching. The proportion of all these fixations toward the target vehicle side of the junction was then calculated. The Proportion of Gaze was calculated in the same way as the previous measure with total gaze duration rather than number of fixations. Total gaze duration is the total time spent on fixations to the target vehicle side of the junction, so Proportion of Gaze gives a general measure of how much visual attention was biased toward the oncoming vehicle. The Mean Fixation Duration was calculated by the total gaze duration toward the target vehicle side of the junction, divided by the number of fixations made toward the target vehicle side of the junction.

**Figure 4.**
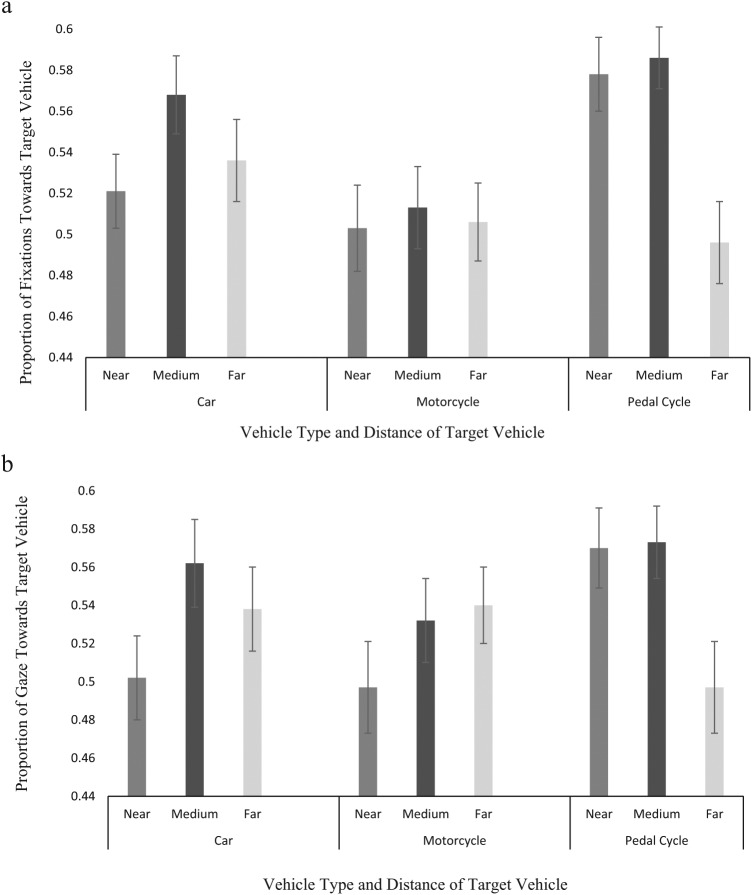
Figure 4a shows the drivers’ proportion of fixations to the target vehicle side of the junction as a function of Vehicle Type and Distance, and Figure 4b shows the drivers’ proportion of gaze as a function of Vehicle Type and Distance. These means are untransformed. Error bars display one standard error above and below the mean.

Proportion of Fixations and Proportion of Gaze were subject to an arcsine transformation due to a leptokurtic distribution. Mean Fixation Duration was subject to a log transformation due to a positive skew in the data. The reported statistics for these measures are from the transformed data. However, Figure 4 shows the untransformed data in order to present proportion units between 0 and 1.

#### Proportion of fixations

A main effect of Vehicle Type was found, *F*(2, 152) = 3.39, MSE = .05, *p* < .05, n^2^_p_ = .04, with contrasts revealing a significant difference between pedal cycles compared with cars and motorcycles, *F*(1, 76) = 3.27, MSE = .05, *p* < .05, n^2^_p_ = .05. Participants had a higher proportion of fixations on the target side of the junction when the approaching vehicle was a pedal cycle compared with a car or a motorcycle.

There was an interaction between Vehicle Type and Distance, *F*(4, 304) = 2.54, MSE = .04, *p* < .05, n^2^_p_ = .03, with contrasts indicating a linear difference between pedal cycles compared with cars and motorcycles, *F*(1, 76) = 7.35, MSE = .06, *p* < .01, n^2^_p_ = .09. Participants’ proportion of fixations toward the target vehicle was greater when cars and motorcycles were approaching from a far distance compared with pedal cycles but greater when pedal cycles approached from a medium or near distance compared with cars and motorcycles (see Figure 4a).

There was also a main effect of Road Sign, *F*(1, 76) = 10.55, MSE = .01, *p* < .01, n^2^_p_ = .12, with participants having a higher proportion of fixations toward the target vehicle when a Stop Sign was present compared with a Give Way Sign. There was no main effect of Group, *F*(1, 76) = .19, MSE = .01, *p* = .66, n^2^_p_ = .01.

#### Proportion of gaze

An interaction between Vehicle Type and Distance was found, *F*(4, 304) = 2.57, MSE = .05, *p* < .05, n^2^_p_ = .03, with contrasts indicating a linear difference between pedal cycles compared with cars and motorcycles, *F*(1, 76) = 7.66, MSE = .08, *p* < .01, n^2^_p_ = .09. Again, participants’ proportion of gaze toward the target vehicle was greater when cars and motorcycles were approaching from a far distance compared with pedal cycles; however, it was greater when pedal cycles approached from a medium or near distance compared with cars and motorcycles (see Figure 4b).

There was also a main effect of Road Sign, *F*(1, 76) = 9.79, MSE = .02, *p* < .01, n^2^_p_ = .11, with participants having a higher proportion of gaze toward the target with a Stop Sign compared with a Give Way Sign. There was no main effect of Group, *F*(1, 76) = .59, MSE = .02, *p* = .45, n^2^_p_ = .01.

#### Mean fixation durations

A main effect of Distance was found, *F*(2, 152) = 4.29, MSE = .05, *p* < .05, n^2^_p_ = .05, with contrasts revealing a significant linear trend, *F*(1, 76) = 3.27, MSE = .05, *p* < .05, n^2^_p_ = .12. Participants had higher mean fixation durations on the target side of the junction when the vehicle was approaching from a closer distance.

There was a main effect of Road Sign, *F*(1, 76) = 11.23, MSE = .15, *p* < .01, n^2^_p_ = .13, with drivers fixating toward the approaching vehicle for longer with a Stop Sign present than a Give Way Sign. There was no main effect of Group, *F*(1, 76) = 1.14, MSE = .15, *p* = .29, n^2^_p_ = .02.

## Discussion

The first immediate finding from the study is that drivers’ attention was not associated with pedal cycling experience at junctions. Despite previous research suggesting that experience with a certain vehicle may change drivers’ visual attention toward this vehicle (Crundall et al., 2012), this does not seem to be the case for pedal cyclists when pulling out of a junction as a driver. The second immediate finding is that, in general, drivers do not look toward pedal cycles approaching from a far distance as much as they look toward motorcycles and cars. In contrast, at closer distances, they may actually look more toward pedal cycles than either motorcycles or cars. Both of these results occurred irrespective of the sign present at the junction, implying that the differences in visual search are present in cases both where the driver generally pulls out in front of the target vehicle and where they wait for it. The manipulation of the change in road sign was conformed to by participants, with drivers pulling out in front of the target vehicle on significantly more occasions with a Give Way Sign present compared with a Stop Sign. It should be noted that these differences have been observed despite the fact that speed of travel for target pedal cycles was identical to that of motorcycles and cars.

In regards to the first finding, although previous literature has established findings indicating that drivers with specific motorcycle experience (Crundall at al., 2012) and pedal cycling experience (Beanland & Hansen, 2017) have more efficient visual attention toward motorcycles and pedal cycles compared with drivers only, this does not seem to be the case when scanning a junction in order to complete a maneuver. This contradictory finding may be explained by the difference in task requirements between our study and previous ones performed by driver-cyclists—passively watching video clips does not require additional demands such as vehicle control and does not require participants to complete a maneuver.

These contradictory results may be a result of the difference between the factors that promote motorcycle and pedal cycle use. Motorcyclists’ views about why and how they ride have been seen to be related to the social context of riding, including social- and identity-related influences relating to the group, as well as self-identity (Tunnicliff, Watson, White, Lewis, & Wishart, 2011). In contrast, cycling may not play such an important role in the self-identity of a cyclist, using a pedal cycle for reasons such as efficiency, flexibility, cost, economy, and health (Levulytė, Baranyai, Török, & Sokolovskij, 2016).

In addition, it may be possible that pedal cyclists are not as aware as motorcyclists about the dangers surrounding junctions. Crash statistics show that motorcyclists are much more likely to be involved in crashes, with motorcycles accounting for 21% of UK road deaths and pedal cyclists accounting for 6% in 2015 (Department of Transport, 2015). Current educational campaigns such as the UK Department for Transport’s Think! Bike are also heavily associated with motorcyclists’ rather than pedal cyclists’ safety. For this reason, it may be the case that pedal cyclists do not have a heightened awareness of the dangers associated with cycling on road compared with that of motorcyclists, which in turn may result in cyclists not having an increased detection of oncoming pedal cyclists compared with that of nonpedal cyclists.

In the absence of differences between pedal cyclists and nonpedal cyclists on all eye movement measures, we conclude that the specific pedal cycling experience of the pedal cyclist group is not associated with changes in drivers’ visual attention toward oncoming pedal cycles or motorcycles at controlled simulated junctions. Given that our sample size provided enough power to detect the effect (medium effect size for within-between interaction, Cohen’s *f* = 0.25, 1-β = 0.80) if it was present, this suggests that pedal cycling experience does not make drivers’ visual attention strategy safer at junctions.

In regards to the second finding, the visual attention measures suggest that drivers do not distribute as much visual attention toward pedal cyclists approaching from far distance compared with motorcycles and cars. It may be the case that drivers do not deem pedal cycles approaching from a far distance to be as dangerous as approaching motor vehicles due to the usual difference in speed and mass and therefore are happy to focus their attention elsewhere in the visual scene for potential danger. It also may be the case that pedal cycles manifest different looming behaviors compared with larger motor vehicles, as faster moving vehicles are thought to loom less than slower vehicles (Wann, Poulter, & Purcell, 2011), and a driver’s ability to detect the motion of an object decreases, the smaller the object is. Although this behavior seems plausible, it is surprising given that the visual parameters of the approaching pedal cycles in this simulation environment, in terms of distance and speed, were identical to the far-approaching motorcycles and cars. This may suggest that drivers are terminating their gaze away from far-approaching pedal cycles too early, not fully forming a representation of the pedal cycle’s speed and distance. It must also be noted that although drivers’ eye movements were aggregated across left- and right-approaching vehicles, drivers’ proportion of fixations (left = .54, right = .51) and proportion of gaze (left = .48, right = .56) did not significantly differ whether the far pedal cycle was approaching from the left or the right.

In addition, drivers approached the junction faster when a pedal cycle was approaching from a near or medium distance compared with a motorcycle or car. These different approach dynamics again suggest that the appraisal of threat from the pedal cyclist may be different, with less time needed to make a decision at the junction. Conversely, drivers’ approach behavior for far-approaching pedal cycles was slower compared with a motorcycle and car, suggesting that drivers took more time to make a decision, possibly due to the perceived low threat of a far-approaching pedal cycle compared with a motor vehicle. Previous research has found that when countermeasures are in place to reduce drivers’ speed on the approach to a junction, this changed drivers’ visual search toward pedal cycles for the better, simply providing more time to look at the approaching vehicle (Summala et al., 1996).

Although pedal cyclists will often be travelling slower than cars or motorcyclists, in urban environments there are plenty of occasions where recreational cyclists can achieve the local speed limit. The current research suggests that such situations may present a particular problem, with drivers failing to pay sufficient attention to distant but relatively fast-moving pedal cyclists. With previous studies demonstrating that drivers are poor at determining the speed of other vehicles, particularly when travelling at high speed (Dommes, Cavallo, Vienne, & Aillerie, 2012), this is particularly relevant to the increase in the use of E-bikes (pedal cycles that provide electrical support). These bikes have been seen to reach higher speeds than conventional pedal cycles; therefore, drivers may misjudge their approaching speed (Schleinitz, Petzoldt, Krems, & Gehlert, 2016).

The subtle differences found in drivers’ everyday visual attention can be used in the development of in-car technologies. As a starting point, this study demonstrates how much visual attention drivers distribute to vulnerable road users dependent on vehicle type and distance at a junction. These eye movement measures can help in the understanding of drivers’ cognitive mechanisms involving the distribution of visual search at a junction in simulated driving environments. The visual information that drivers obtain at junctions, which inevitability informs their behavior, is important for the development of in-car technical assistive systems for drivers, making drivers safer. One particular source of information that may be important for such systems to provide would be warnings related to distant pedal cycles that are nonetheless approaching the driver at relatively high speeds. In regards to road safety, this in turn could help prevent the high proportion of crashes at junctions involving these road users.

Finally, it must be noted that no crashes occurred during the experiment. Although many crashes between cars and either pedal cycles or motorcycles do occur at real junctions, it is important to remember that these crashes are nonetheless rare events—the vast majority of real junction crossings are conducted safely. Even though we observed 1,440 experimental trials in the course of this study, this is still relatively little driving compared with the expected frequency of crashes on real British roads (less than one crash per 10,000 miles of driving; Department of Transport, 2016). We did explore safety margins by measuring the shortest time to contact with an oncoming vehicle on occasions where the driver chose to pull out, but we found that this did not differ overall as a function of the oncoming vehicle type. In terms of specific “near crash” events, defined by a time to contact of below 2 seconds (e.g., Matsui, Takahashi, Imaizumi, & Ando, 2011), it was found that there were marginal, Cochran’s Q(2) = 5.20, *p* = .074, *n* = 80, differences in the frequency of “near crashes” as a function of oncoming vehicle type—pedal cycle (4 participants), motorcycle (1 participant), and car (0 participants). This tendency, combined with the relatively high approach speeds for near and medium pedal cycles, provides support for the idea that drivers may be more likely to “take a chance” in front of a pedal cyclist than an oncoming motorcycle or car, even though the approach speeds are matched.

In conclusion, this study provides important and novel information, indicating that drivers who pedal cycle frequently do not show any differences in their visual attention toward pedal cycles on the road compared with nonpedal cyclists, despite previous research finding this in other settings. We found that drivers do not distribute as much visual attention toward pedal cycles approaching from a far distance despite them approaching at identical speeds to motorcycles and cars. These subtle differences in drivers’ visual attention shed light on drivers’ everyday visual search at junctions as a function of vehicle type, which can have important implications for vulnerable road users’ safety.

## Key Points

- Previous studies have found that specific motorcycle experience enhances drivers’ visual attention toward motorcycles at junctions; however, no previous research has investigated the effect pedal cycling experience has on drivers’ visual attention toward pedal cycles at junctions, despite the increase in pedal cycle use on public roads.
- Drivers’ visual attention at junctions was not associated with pedal cycling experience in a simulation environment.
- Drivers, in general, do not distribute as much attention toward pedal cycles approaching from far distances, despite them approaching at identical speeds to cars and motorcycles.
- Subtle differences in drivers’ visual attention toward vulnerable road users at junctions are important for the future safety of these road users.
